# Caudal Clonidine in Day-Care Paediatric Surgery

**Published:** 2009-08

**Authors:** Archna Koul, Deepanjali Pant, Jayshree Sood

**Affiliations:** 1 Consultant, Deptt of Anaesthesiology, Pain and Perioperative medicine, Sir Gangaram Hospital, New Delhi; 2 Asso. Consultant, Deptt of Anaesthesiology, Pain and Perioperative medicine, Sir Gangaram Hospital, New Delhi; 3 Senior consultant and Chairperson, Deptt of Anaesthesiology, Pain and Perioperative medicine, Sir Gangaram Hospital, New Delhi

**Keywords:** Caudal epidural, Clonidine, Bupivacaine

## Abstract

**Summary:**

We evaluated the analgesic efficacy, hemodynamic and respiratory safety of Clonidine when added to bupivacaine for caudal block. Forty children undergoing inguinal hernia repair were randomly given caudal injection with 0.75 ml.kg^−1^ of bupivacaine (0.25%) and clonidine 2 μg.kg^−1^ in Group C or 0.75 ml.kg^−1^ of bupivacaine (0.25%) alone in Group B after induction of anaesthesia. Postoperatively duration of analgesia, OPS score (observational pain / discomfort scale), Sedation score, heart rate and blood pressure were recorded. Duration of analgesia was significantly longer (p<0.001) in Group C (10.25 hours) as compared to 4.55 hours in Group B. Bradycardia, hypotension and sedation were not observed in Group C. The addition of Clonidine in caudal blocks prolongs postoperative pain relief in children and is safe alternative to bupivacaine alone in paediatric daycare surgeries.

## Introduction

Caudal epidural analgesiais one of the most popular regional blocks inpaediatric anaesthesia, but its main disadvantage is short duration of action after single injection (4-6hrs) even with a long acting local anaesthetic.

Prolongation of caudal block using a single shot technique; can be achieved by addition of various adjutants. Epinephrine was the earliest adjuvant drug used, during second half of 1980's. Opioids were also used but their use carried risk of respiratory depressions^1^. Ketamine as an adjutant has potential for neurotoxic effects if in advertently injected intrathecally^2^ and neostigmine is accompanied by 30% incidence of vomiting^3^.

Clonidine, an α-2 adrenergic agonist, produces analgesia without significant respiratory depression. The analgesic action of epidurally administered Clonidine is due to stimulation of descending noradrenergic medullospinal pathways inhibiting the release of nociceptive neurotransmitters in the dorsal horn of spinal cord^4^. The analgesic effect of Clonidine is more pronounced after neuraxial injection which suggests a spinal site of action and makes this route of administration preferable^5^,^6^.

We designed a prospective randomized double blind study to examine the haemodynamic and respiratory effects of clonidine as an adjunct to bupivacaine caudal epidural analgesia and compare its analgesic efficacy with control group in which only bupivacaine was used, in paediatric day care procedures.

## Methods

After informed parental consent, 40 ASAI children between 1-10 years of age weighing 5-20 kg, who were scheduled to undergo inguinal herniotomy as daycare cases were admitted in the study. Children with any contraindication to caudal block or having congenital heart disease were excluded from the study. No premedication was given and all surgeries were performed under general anaesthesia. Inhalational induction was done with oxygen, nitrous oxide and 8% sevoflurane delivered through Jacks on Rees modification of Ayre's T-piece and face mask, with standard monitoring. After intravenous cannula insertion a PUMA (Proseal Laryngeal Mask Airway LMA company Ltd.) of appropriate size (between size 1- 1.5), depending on the weight of the patient, was introduced. Anaesthesia was maintained with 33% oxygen, 67% nitrous oxide and sevoflurane (3-4%) with manual assistance and fresh gas flow of 2-3 litres /minute. After induction of anaesthesia, the children were assigned randomly to receive a caudal ep idural injection of either 0.75 ml.kg^−1^ of 0.25% bupivacaine (Group B) or 0.75 ml.kg^−1^ of 0.25% bupivacaine with 2μg.kg^−1^ of clonidine (Group C). Clonidine was loaded in 1 ml syringe so as to keep the volume same in both the groups. Caudal block was performed with 23 gauge hypodermic needle under aseptic conditions in left lateral position and the child was turned supine after the injection of the drug. Mean blood pressure and heart rate were recorded before induction of anaesthesia and every 5 minutes subsequently. An intraoperative increase in heart rate and blood pressure by more than 10% after 15 minutes of caudal injection was defined as insufficient analgesia and treated with intravenous fentanyl lmcg.kg^−1^.

At the beginning of skin closure, sevoflurane and nitrous oxide were discontinued. When the children were sufficiently awake, PLMA was removed and they were shifted to the recovery room, breathing room air.

After arrival mean blood pressure, heart rate and SpO_2_ was documented hourly for 6 hours. Intraoperative or postoperative decrease in mean blood pressure or heart rate more than 30% of the baseline values was defined as severe hypotension or bradycardia respectively. Respiratory depression was defined as a decrease of SpO_2_ less than 93% and required supplemental oxygen viamask. Pain assessment in the post-operative period was done by an observer blinded to the therapeutic intervention using OPS score (at 30 minutes, 1 hour and 2 hours) and duration of analgesia. Each variable (crying, facial expression, verbal response, position of torso and motor restlessness) was scored between 0-2 (0: none, 1: moderate, 2: severe), to give a cumulative score of 0-10. Ifthe OPS score was more than 4 in 2 subsequent measurements or ifthe patient showed obvious signs of pain they were given oral paracetamol 10 mg.kg^−1.^ The duration of postoperative analgesia was defined as time between caudal drug injection and the first complaint of pain. Assessment of sedation was done by sedation score at 30 minutes, l hour and 4 hours after the operation. Patient sedation score was defined as 1: asleep, not arousable by verbal contact; 2: asleep, arous able by verbal contact; 3: drowsy not sleeping; 4: alert/aware. This helped in identification of respiratory depression. Duration of motor blockwas assessed by noting the time children began moving their legs. Time of first micturition was also noted. Children were discharged from the daycare ward after 6 hours of arrival. Parents noted the total requirement of additional analgesia in first 24 hours after operation on a form and were contacted telephonically.

Parametric data was analysed using Student's t test.

## Results

**Fig 1:**
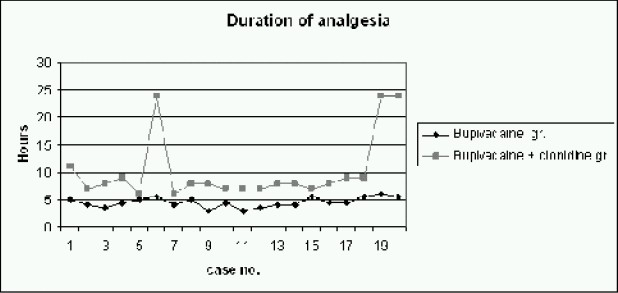
Comparison of duration of analgesia in hours between both groups

Mean age, weight, surgical and recovery time were comparable between two groups (mean age of Group B: 3.3 years, mean age of Group C: 3.5 years, mean weight of Group B: 13.5 kg, mean weight of Group C: 14.6kg, mean surgical time of Group B: 39.7 min, mean surgical time of Group C:43.8 min, as shown in Table 1). The duration of analgesia and the time for first postoperative analgesic requirement was significantly prolonged in Group C (Table 2 & Fig 1). There was a decrease from base line mean arterial pressure in Group C 5-15 minutes after the injection but it returned to normal in 30 minutes. The mean time for first analgesic requirement in Group C was 10.25 ± 6.03 hours, while it was 4.5 ± 0.87 hours in bupivacaine Group (Table 2 & Fig 1).

**Table 1:** Demographic distribution

	GrB	GrC
Age(years)	3.28±1.65	3.45±2.06
Weight(kg)	13.45±6.04	14.58±4.40
Duration of surgery(min)	41±13.19	43.75±12.23

**Table 2:** Shows mean values with standard deviation of both groups and respective p-values

	GrB	GrC	p-value
Intraop MAP(mmHg)	62.2±11.23	62.8±10.07	0.86
Postop MAP(mmHg)	65.6±9.37	70.15±10.09	0.15
Intraop HR(bpm)	102.35±8.65	102.3±10.14	0.99
Postop HR(bpm)	95.4 ±6.8	97.05±9.97	0.54
Duration of analgesia(hr)	4.5±.87	10.25±6.03	0.00
Mean sedation score	2.8±0.45	2.83±0.47	0.84
Mean OPS score	4.65±0.25	4.55±0.25	0.62

**Fig 2:**
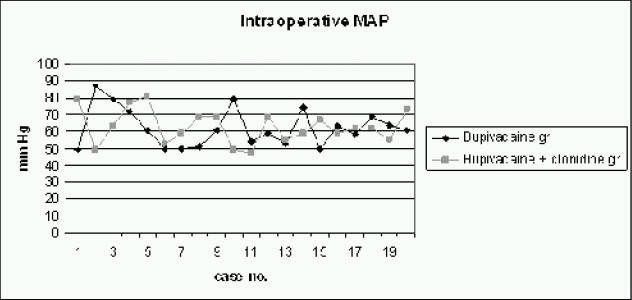
Comparison of Intraoperative Mean Arterial Pressure in mm Hg between both groups

**Fig 3:**
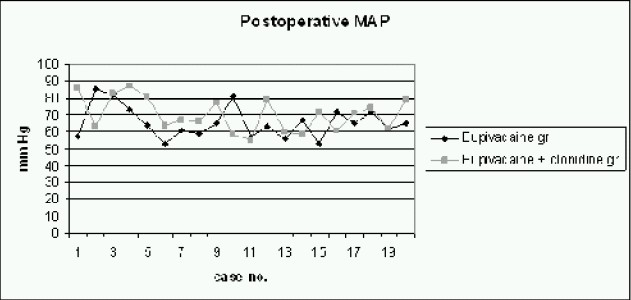
Comparison of Postoperative Mean Arterial Pressure in mm Hg between both groups

The comparison of mean arterial pressure during intraoperative and post operative period (Fig 2 & 3), between both groups, using Student's t test, yielded p-values (0.86 for intraoperative MAP and 0.15 for post-operative MAP) which were not significant. The comparison of heart rate during intraoperative and post operative period, between both groups showed insignificant p-value (p-value for intraoperative heart rate was 0.99 and forpostoperative heart rate it was 0.54). With N as 20 in each group with a m can prolongation of analgesia by 5.75 hours in Group C as compared to Group B (Fig 6), the power of study was 94.2% and the p-value less than 0.05 (p<0.001).

**Fig 4:**
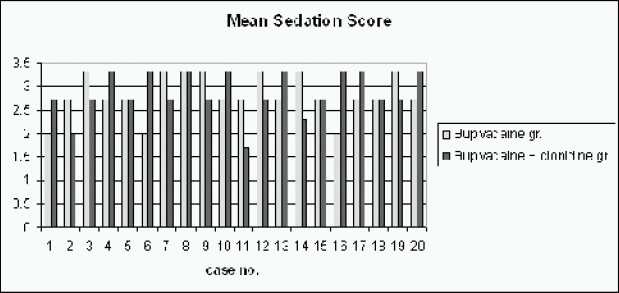
Mean sedation score in both groups

**Fig 5:**
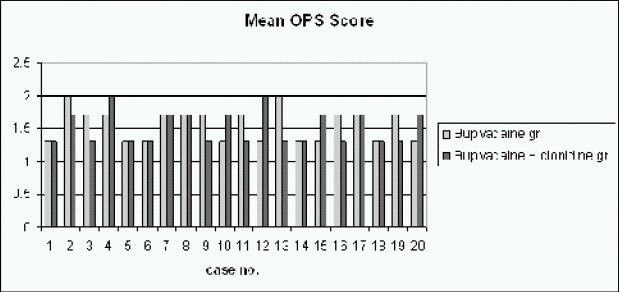
Mean OPS score in both groups

None of the children had urinary retention and the incidence of vomiting was comparable between the groups (Table 1,2). None of the children had SpO_2_ value less than 95%. None of the children had bradycardia. On awakening no motorblock was observed in any case in either group. There was no significant difference in either group in the mean postoperative sedation score (Group B was 2.8± 0.45 and Group C was 2.83, (Table 2 & Fig 4). The mean OPS score of Group B was 4.65 ± 0.25 and Group C was 4.55 f 0.25 (Table 2 & Fig 5). The mean time of firstmicturition in Group B was 4.8 hours and in Group C was 4.7 hours.

**Fig 6:**
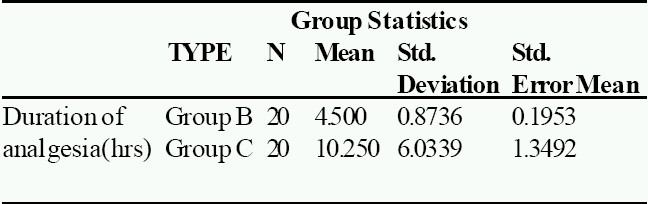
T-Test for Duration of Analgesia

## Discussion

Our data indicates that for caudal blockade, the addition of clonidine 2 μg.kg^−1^ with 0.25% bupivacaine significantly prolongs the duration of analgesia (Table 2 & Fig 1). This reduces the need for both intraoperative and postoperative analgesia. These findings confirm with a previous study^7^ which reported that 1-2μg of clonidine with bup ivacaine increases duration of analgesia in small children undergoing day care hernia repair compared to bupivacaine alone or bupivacaine with epinephrine 5μg.ml^−1^.

Duration of analgesia in this study is less than that observed in another study^8^ where they used combination of ketarnine 1 mg.kg^−1^ and clonidine 1-2 μg.kg^−1^ of caudal blockade in children.

Our experience with clonidine for caudal blockade is consistent with the results obtained from other studies^9^‐^11^, where clondine 1-2 μg.kg^−1^ was added to caudal bupivacaine for supraumbilical, urn logical or orthopaedic surgeries.

The undesirable side effects ofneur Gaxial clonidine are hypotension and bradycardia. Many studies^6^,^12^,^13^ in adult patients have reported adecrease in MAP and HR within 15-30 minutes after ep idural injection which lasted for 3-4 his before returning to baseline. In our study decrease in blood pressure did occur in all patients who were given clonidine with bupivacaine within 5-15 minutes but returned to normal within 30 minutes.

Some studies^12^,^13^ report a marked decrease in heart rate afler epidural clonidine while others do not. We did not observe any significant bradycardia in our study.

Dose dependent sedation usually accompanies the use of clonidine for regional anaesthesia^6^,^14^ however with 2μg.kg^−l^, we did not appreciate any excessive sedation (Fig 2 and 6). All the children were sleeping but arousable andthis was appreciated by the parents.

All children could move their legs at the end of 6 hour period and there was no case of retention of urine. Except fort patients, there was no case of postoperative vomiting and therefore discharge of no child was delayed.

This study used patients undergoing the same surgical procedure, no premeditation and same pattern of induction so as to eliminate any factor that could have interfered with analgesia requirement or haemodynamic variables.

The use of parents to assess pain after discharge till 24 hours may introduce some sort of inconsistency in deciding to medicate the child but it is the parents who know their child best.

The main purpose of our study was to assess the suitability ofclonidine and bupivacaine in daycare patients who are to go home the same day. Absence of any side effect like sedation, bradycardia, vomiting and respiratory depression with reduction in the need of analgesic goes in the favor of using clonidine as an adjuvant with local anaesthetic in this set of patients.

We chose dose of 2μg.kg^−1^ as compared to 1μg.kg^−1^ as used in many studies, as this dose provides benefit to the child with no additional risk of side effects.

This combination of 2 μg.kg^−1^ clonidine with bupivacaine 0.25% is a safe and effective alternative to bupivacaine alone for caudal block in peadiatric day care surgeries.
